# Integrative Processing of Touch and Affect in Social Perception: An fMRI Study

**DOI:** 10.3389/fnhum.2016.00209

**Published:** 2016-05-10

**Authors:** Sjoerd J. H. Ebisch, Anatolia Salone, Giovanni Martinotti, Leonardo Carlucci, Dante Mantini, Mauro G. Perrucci, Aristide Saggino, Gian Luca Romani, Massimo Di Giannantonio, Georg Northoff, Vittorio Gallese

**Affiliations:** ^1^Department of Neuroscience, Imaging and Clinical Sciences and Institute of Advanced Biomedical Technologies, G. d’Annunzio University of Chieti-PescaraChieti, Italy; ^2^Department of Psychological, Health and Territorial Sciences, School of Medicine and Health Sciences, G. d’Annunzio University of Chieti-PescaraChieti, Italy; ^3^Department of Health Sciences and Technology, ETH ZurichZurich, Switzerland; ^4^Department of Experimental Psychology, University of Oxford, OxfordUK; ^5^Research Center for Motor Control and Neuroplasticity, KU LeuvenLeuven, Belgium; ^6^The Royal’s Institute of Mental Health Research & University of Ottawa Brain and Mind Research Institute, Centre for Neural Dynamics, Faculty of Medicine, University of OttawaOttawa, ON, Canada; ^7^Section of Physiology, Department of Neuroscience, University of ParmaParma, Italy; ^8^Institute of Philosophy, School of Advanced Study, University of LondonLondon, UK

**Keywords:** social perception, emotion, somatosensory, fMRI, facial expression, tactile sensation

## Abstract

Social perception commonly employs multiple sources of information. The present study aimed at investigating the integrative processing of affective social signals. Task-related and task-free functional magnetic resonance imaging was performed in 26 healthy adult participants during a social perception task concerning dynamic visual stimuli simultaneously depicting facial expressions of emotion and tactile sensations that could be either congruent or incongruent. Confounding effects due to affective valence, inhibitory top–down influences, cross-modal integration, and conflict processing were minimized. The results showed that the perception of congruent, compared to incongruent stimuli, elicited enhanced neural activity in a set of brain regions including left amygdala, bilateral posterior cingulate cortex (PCC), and left superior parietal cortex. These congruency effects did not differ as a function of emotion or sensation. A complementary task-related functional interaction analysis preliminarily suggested that amygdala activity depended on previous processing stages in fusiform gyrus and PCC. The findings provide support for the integrative processing of social information about others’ feelings from manifold bodily sources (sensory-affective information) in amygdala and PCC. Given that the congruent stimuli were also judged as being more self-related and more familiar in terms of personal experience in an independent sample of participants, we speculate that such integrative processing might be mediated by the linking of external stimuli with self-experience. Finally, the prediction of task-related responses in amygdala by intrinsic functional connectivity between amygdala and PCC during a task-free state implies a neuro-functional basis for an individual predisposition for the integrative processing of social stimulus content.

## Introduction

The recognition of others’ emotions and bodily feelings offers primary information to predict and attribute meaning to the intentional behavior of others. One important and outstanding question concerns the integrative processing of such signals from our social world in order to give sense to complex social perceptions. Social perception commonly employs multiple sources of information regarding others’ experiences. For example, at a primary non-verbal level, a strong link exists between emotional expressions and bodily sensations as well as motor behavior, while the latter two are often used to infer others’ affective states (Gallese et al., 2004; Bastiaansen et al., 2009). How is the integration of manifold bodily signals accomplished in the human brain?

An impressing amount of neuroscientific and meta-analytic data suggests that these different types of information recruit partially distinct functional networks, including sensorimotor, affective and mentalizing circuits (Gallese, 2003; Van Overwalle and Baetens, 2009; Keysers et al., 2010; Bernhardt and Singer, 2012; Bickart et al., 2014; Schurz et al., 2014). However, also overlap has been reported in sensorimotor and affective circuits supporting the social perception of both others’ sensorimotor and others’ affective experiences (Molenberghs et al., 2012) as well as between pre-reflective and inferential forms of social cognition (Fan et al., 2011). Except for affective regions (e.g., anterior insula, anterior cingulate cortex, and amygdala), also sensorimotor structures contributing to the recognition of others’ bodily sensations (e.g., somatosensory cortex, frontal operculum, and premotor cortex; Keysers et al., 2010; Gallese and Ebisch, 2013) are involved in recognizing others’ affective states (Adolphs et al., 2000; Pitcher et al., 2008; Hillis, 2014). Brain regions more generally involved in different types of social information processing could be hypothesized to have integrative functions, like conflict resolution or affective responses based on coherence of information from multiple sources (Etkin et al., 2006; de Lange et al., 2008; Muller et al., 2011).

Some studies provided insight into the neural mechanisms that may contribute to the integrative processing of social information from multiple sources. For instance, supramodal representations of crossmodal information (visual and auditory information) about others’ emotional states have been linked with left amygdala and posterior cingulate cortex (PCC), whereas ambiguous crossmodal information elicited stronger neural activity in a network comprising frontoparietal sensorimotor and cingulo-insular affective circuits (Klasen et al., 2011). Furthermore, by studying contextual framing of social signals, stronger activity was found in bilateral amygdala, anterior insula, temporal pole, and fusiform gyrus for facial expressions of emotion in affective contexts compared with neutral contexts (Mobbs et al., 2006). However, it remains poorly understood whether these circuits could be involved more specifically in the integrative processing of social information about others’ feelings from manifold bodily sources (e.g., “direct” affective information from facial expressions and “indirect” affective information from sensorimotor experiences). Modulating the coherence of social stimulus content (e.g., directly comparing congruent and incongruent information) within a single domain (e.g., unimodal, visual, information) could offer the possibility to study brain integrative functioning at the basis of making sense of the content of our social perceptions.

Interestingly, amygdala and fusiform gyrus are involved together in face perception (Adolphs, 2002; Herrington et al., 2011). Amygdala has been associated particularly with encoding relevance and impact of socio-emotional stimuli including faces (Ewbank et al., 2009; Adolphs, 2010; Bickart et al., 2014), and subjective judgments of facial expressions of emotions (Adolphs et al., 2002; Wang et al., 2014). In addition, PCC supports self-related processing by integrating external stimuli in one’s own personal context through the interaction between memory and emotion (Northoff and Bermpohl, 2004; Vogt et al., 2006; van der Meer et al., 2010), whereas anterior insula associates self-related processing with the organisms transient physiological bodily states (Craig, 2009). Hence, these brain structures could contribute to the integrative processing of social information underlying the awareness of others’ affective experiences in complex social perceptions. This possibly is mediated by the self-relatedness of content (Vogt et al., 2006; Northoff et al., 2009; Bickart et al., 2014). In particular, congruent content or social perceptions likely is more familiar to the observer in terms of own personal experiences, leading to a higher self-relatedness or relevance.

The present functional magnetic resonance imaging (fMRI) study aimed at elucidating the integrative processing of multiple signals during social perception in the visual domain, focusing on facial expressions of emotion of other individuals that are either congruent or incongruent with the tactile sensations of those individuals. Both are frequently employed bodily sources to understand another one’s inner state, but are rarely investigated simultaneously. Moreover, the relationship between task-evoked neural responses and ongoing brain activity during a task-free state will be investigated. The latter could clarify how neural responses to complex social stimuli depend on intrinsic brain function which is proposed to constitute a neural predisposition characterizing individual reactions to external stimuli (Northoff, 2013, 2014).

It was hypothesized that the encoding of social stimulus congruence depends on brain circuits underlying affective and self-related processing, like amygdala, fusiform gyrus, anterior insula, and PCC. Increased neural responses in these structures imply enhanced integrative processing of others’ affective experiences due to coherence of content. Alternatively, but not mutually exclusive, conflicting information could claim higher neural processing capacity in sensorimotor and affective circuits or in the mentalizing network associated with inferential/effortful social cognition. Finally, it was expected that enhanced neural responses to congruent stimuli could be associated with task-free brain activity as measured by intrinsic functional connectivity patterns of the implied brain structures, especially regions associated with high metabolism during task-free states and self-related processing, like PCC.

## Materials and Methods

### Participants

Twenty-six participants were included in the present fMRI study (five females, age range: 20–42 years). All participants were healthy, right handed and had normal vision capabilities (correction < 0.75). Written informed consent was obtained from all participants after full explanation of the procedure of the study, in line with the Declaration of Helsinki. The experimental protocol was approved by the local institutional ethics committee. The participants were given a recompense for participating in the fMRI experiment.

### Stimuli

Four types of video stimuli were created for the experiment. To control for effects due to the integrative processing of information from different perceptual modalities rather than content, participants in the fMRI experiment were presented social information only in the visual domain: short video clips depicting others’ sensory-affective experiences.

**FIGURE 1:**
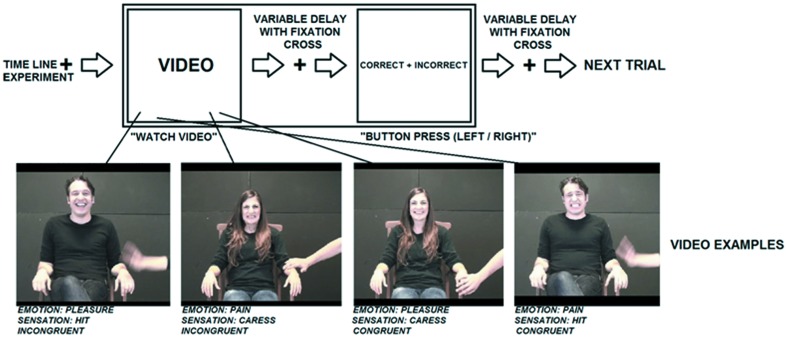
**Visualization of the experimental stimuli and procedure**.

The duration of each video clip was 2400 ms. Video clips were in color and depicted an actor sitting on a chair and being caressed or hit on their left hand by another actor. While being caressed or hit, the actor facially expressed an emotion, pleasure or pain, that could be either congruent or incongruent with the sensation induced by the touch. In half of the clips, a woman expressed an emotion, while being caressed or hit by a man. In the other half a man expressed an emotion, while being caressed or hit by a woman. Of the touching actor only the hand and arm were visible. These video clips can be categorized in four experimental conditions based on the combination of tactile sensation and facial expression: (1) caress-pleasure, (2) hit-pain, (3) caress-pain, and (4) hit-pleasure. Videos stills are visualized in **Figure 1**.

To evaluate whether the congruent and incongruent video stimuli used for the fMRI experiment differed with respect to the perceived self-relatedness and familiarity with the content, an independent sample of 42 participants (31 males, 11 females; 20–37 years) was included in the study (after concluding the fMRI experiments) and asked to judge these aspects of the stimuli. Chi-square tests showed that there was no significant difference in gender distribution between the sample included in the fMRI experiment and the stimulus judgments (χ^2^ = 0.43, *p* > 0.5). Since different participants were included in the fMRI task and the stimulus ratings, the results obtained by both procedures cannot be explained by an interaction between them, possibly altering stimulus interpretation: the fMRI results (stimulus congruence coding) could not be influenced by the self-relatedness/familiarity judgments, and the self-relatedness/familiarity judgments could not be influenced by the fMRI task (stimulus congruence coding).

Firstly, self-relatedness was assessed by asking participants “How much do you personally associate with or relate to this picture?” (translated from Italian). It was further explained that they needed to rate the personal association with the video content based on the strength of their subjective or personal experience of themselves while viewing the videos (see also Schneider et al., 2008). Secondly, to assess participants’ familiarity with the video content, all videos were shown again and participants were asked “How much are you familiar with the experience depicted by the video” (translated from Italian). To indicate the degree of self-relatedness or familiarity, participants’ responses were obtained by a drawing a horizontal line on a Visual Analog Scale (vertical line of 10 cm) ranging from “low personal association” to “high personal association” or from “low familiarity” to “high familiarity,” respectively. Ratings were quantified by measuring the distance in mm between the lower point of the line and the sign of the participant on the scale.

Self-relatedness judgments yielded the following ratings: caress-pleasure = 56.59 ± 25.38; hit-pain = 40.25 ± 28.22; caress-pain = 23.35 ± 20.41; hit-pleasure = 38.72 ± 22.55. Analysis of variance (ANOVA) concerning the self-relatedness judgments of the video stimuli showed a significant interaction between facial expression and tactile sensation (*F*_1,41_ = 17.149, *p* < 0.001), indicating an effect of stimulus congruence. *Post hoc* analysis showed that the caress-pleasure condition (congruent) was characterized by a stronger self-relatedness than the hit-pleasure condition (incongruent; *p* < 0.001) and the caress-pain condition (incongruent; *p* < 0.005). The hit-pain condition (congruent) was characterized by a stronger self-relatedness than the caress-pain condition (incongruent; *p* < 0.005), while there was no difference between the hit-pain condition (congruent) and hit-pleasure condition (incongruent; *p* > 0.5). In addition to the interaction effect, a main effect of facial expression was found due to a stronger self-relatedness of the expression of pleasure, compared to pain (*F*_1,41_ = 19.614, *p* < 0.001). No significant effect of tactile sensation was found on self-relatedness (*p* > 0.5).

Familiarity judgments yielded the following ratings: caress-pleasure = 63.73 ± 25.13; hit-pain = 34.72 ± 28.51; caress-pain = 24.02 ± 17.99; hit-pleasure = 38.71 ± 25.06. ANOVA concerning the familiarity judgments of the video stimuli showed a significant interaction between facial expression and tactile sensation (*F*_1,41_ = 25.740, *p* < 0.001), indicating an effect of stimulus congruence. *Post hoc* analysis showed that the caress-pleasure condition (congruent) was characterized by a stronger familiarity than the hit-pleasure (incongruent; *p* < 0.005) and the caress-pain condition (incongruent; *p* < 0.001). The hit-pain condition (congruent) was characterized by a stronger familiarity than the caress-pain condition (incongruent; *p* < 0.005), while there was no difference between the hit-pain condition (congruent) and the hit-pleasure condition (incongruent; *p* > 0.5). In addition to the interaction effect, a main effect of facial expression was found due to a stronger self-relatedness of the expression of pleasure, compared to pain (*F*_1,41_ = 35.436, *p* < 0.001), and a main effect of tactile sensation was found due to a stronger familiarity of caress, compared to hit (*F*_1,41_ = 4.716, *p* < 0.05).

### Experimental Procedure

The participant was in a supine position in the MRI scanner and completed two task-free fMRI blocks (2 min × 5.2 min; eyes open with fixation cross) and four task fMRI runs (4 min × 7.7 min). During task fMRI, participants were shown a series of brief video clips as described above. The conditions were presented in randomized order and each condition (type of stimulus) was repeated 48 times.

The experimental conditions allowed investigating the independent effects of tactile sensation, facial expression and their interaction (i.e., effect of congruence between tactile sensation and facial expression). Importantly, regarding the sensation × expression interaction effect, the content of the videos was exactly the same between the congruent and incongruent conditions (conditions 1 and 2 *versus* conditions 3 and 4).

Each video clip was followed by a fixation cross with a random duration of 2400, 4800, or 7200 ms. In 23% of the cases (*N* = 48) and in casual order, the fixation cross was followed by the question: “Please indicate by means of a button press whether you find that the emotional expression (pleasure, pain) in the last video was correct (i.e., congruent) or incorrect (i.e., incongruent) with respect to the tactile sensation (caress, hit).” This task made it possible to direct and monitor the attention of the participant to both the facial expression and the tactile sensation depicted by the video clips. Furthermore, although this task required an explicit judgment of the tactile sensation and facial expression, and their congruency, it avoided forced choices based on conflicting information, because participants were not asked to make decisions about the actor’s experience. For instance, it was not asked to decide “how does the actor depicted in the video feel considering the sensation and expression together,” since this may enhance conflict processing when the expression is incongruent with the sensation.

Since it was not predictable when the questions would appear, participants needed to attend both aspects in all videos to be able to respond correctly when required. Specifically, when participants were required to respond, the words “correct” and “incorrect” appeared on the left and right side of the screen for 2400 ms. Participants were asked to press either the left or right button with the index or medium finger of their right hand. In order to avoid that participants could predict and prepare an eventual motor response with a particular finger, the words “correct” and “incorrect” were presented randomly in left–right or right–left order. For example, if the last seen video was congruent and “correct” was written on the left side, while “incorrect” appeared on the right side, participants responded with a left button press with their index finger. Differently, if the last seen video was congruent and “correct” appeared on the right side, participants responded with a right button press with their medium finger.

The time course of the experiment is visualized in **Figure 1**. Prior to scanning, participants underwent a practicing session outside the scanner to assure that they understood the task.

### fMRI Data Acquisition

For each participant, blood-oxygen-level-dependent (BOLD) contrast functional imaging was performed with a Philips Achieva scanner at 3 Tesla at the Institute of Advanced Biomedical Technologies (ITAB), Chieti, Italy. A T1-weighted anatomical (3D MPRAGE pulse sequence; 1 mm isotropic voxels) and T2^∗^-weighted functional data were collected with an eight channel phased array head coil. Two task-free, eyes-open (fixation cross) scanning blocks were performed consisting of 160 functional volumes each. Four task-fMRI scanning blocks were performed consisting of 193 functional volumes each. EPI data (gradient echo pulse sequence) were acquired from 33 slices (in-plane voxel size 2.396 mm × 2.396 mm, slice thickness 3.5 mm), TR = 2400 ms, TE = 72 ms, flip angle = 80°, Field of View = 230 mm). Slices were oriented parallel to the AC–PC axis of the observer’s brain.

### fMRI Data Preprocessing and Analysis

Raw fMRI data were analyzed with Brain Voyager QX 2.3 software (Brain Innovation, Maastricht, The Netherlands). Due to T1 saturation effects, the first five scans of each run were discarded from the analysis. Preprocessing of functional data included slice scan time correction, motion correction and removal of linear trends from voxel time series. A three-dimensional motion correction was performed with a rigid-body transformation to match each functional volume to the reference volume estimating three translation and three rotation parameters. Preprocessed functional volumes of a participant were co-registered with the corresponding structural data set. As the 2D functional and 3D structural measurements were acquired in the same session, the coregistration transformation was determined using the slice position parameters of the functional images and the position parameters of the structural volume. Structural and functional volumes were transformed into the Talairach space (Talairach and Tournoux, 1988) using a piecewise affine and continuous transformation. Functional volumes were re-sampled at a voxel size of 3 mm × 3 mm × 3 mm and spatially smoothed with a Gaussian kernel of 6 mm full-width half maximum to account for intersubject variability.

The task-fMRI time series were modeled by means of a two gamma hemodynamic response function using predictors (videos differentiated by experimental condition and question/response). The intertrial interval was used as a baseline period and was not modeled. Prior to statistical analysis, a percent signal change normalization of the time series from the different runs was performed. The parameters (beta values) estimated in individual subject analysis were entered in a second level voxel-wise random effect group analysis.

The following effects were tested by an ANOVA: (1) within-subject factor “facial expression (pain, pleasure)” [(caress-pleasure + hit-pleasure) *versus* (caress-pain + hit-pain)]; (2) within-subject factor “tactile sensation (hit, caress)” [(caress-pleasure + caress-pain) *versus* (hit-pain + hit-pleasure)]; (3) interaction effect “facial expression × tactile sensation” [(caress-pleasure + hit-pain) *versus* (caress-pain + hit-pleasure)]. Note that the interaction effect is equivalent to the contrast between the congruent and the incongruent conditions and, thus, indicating the congruence effect. The *p*-value (<0.001 uncorrected) of the group statistical maps and an estimate of the spatial correlation of voxels were used as input in a Monte Carlo simulation (1000 simulations) to access the overall significance level and to determine a cluster size threshold (k) in order to obtain a significance level that was cluster level corrected for multiple comparisons (*p* < 0.05 corrected; *k* > 10, *F* > 13.88, and *p* < 0.001 at the voxel level; Forman et al., 1995; Cox, 1996).

### Covariance Structural Equation Modeling (SEM)

Complementary to the principal analysis of task-evoked BOLD responses, structural equation modeling (SEM) was applied as a confirmatory method to infer task-related functional interactions between brain regions from task-related activations within brain regions. In particular, it allows to test specific hypotheses about functional dependence of activity patterns between brain regions, in our case during the social perception of congruent *versus* incongruent social stimuli across participants.

Structural equation modeling conveys assumptions about the relationships between activity in brain regions in terms of uni- or bi-directional interaction effects by combining anatomical connectivity information and functional data of covariance across participants. Different from PsychoPhysical Interactions (Friston et al., 1997), it is model-based and allows more complex models that consider multiple brain regions and interactions. Different from Dynamic Causal Modeling (DCM; Penny et al., 2004), SEM is a static model, and is not directly influenced by variations and shape of hemodynamic responses (Handwerker et al., 2012). Although, the same analysis on a larger number of subjects is recommended to draw final conclusions on the nature of amygdala interactions suggested by SEM, satisfying reliability of SEM results has been demonstrated for a sample size typical for fMRI studies (Protzner and McIntosh, 2006). Differential beta-values for the contrast between the congruent and the incongruent conditions were used as extracted from the regions of interest (ROIs) characterized by a significant tactile sensation × facial expression interaction effect.

Prior to SEM, an exploratory factor analysis [Principal Axis Factoring (PAF)] was performed on the differential beta-values (congruent minus incongruent conditions) from the interaction ROIs obtained by the voxel-wise ANOVA. PAF allows to select a set of ROIs offering a good compromise between model complexity and interpretability for further SEM. Specifically, relying on the same statistical information (i.e., covariance) as SEM, PAF identifies a latent component: a “hidden” variable inferred from the correlations between the observed activation patterns in the ROIs through a mathematical model. As such, a factor obtained by PAF highlights a discrete network of selected ROIs characterized by common activation patterns suggesting functional interaction among them, though not providing any information about directionality. According to the scree test (Cattell, 1966), one factor could be extracted if explaining 49.61% of the variance, whereas absolute loadings can be required for each ROI greater than 0.30 (Kline, 1994). PAF yielded a satisfying one-factor solution including five ROIs with above threshold loadings exclusively on the first factor: left fusiform gyrus (FFG), left dorsal PCC, bilateral ventral PCC and left amygdala (**Table 1**).

**Table 1:** Pattern matrix of the PAF analysis.

	*Factor*		
	1	2	3
Left fusiform gyrus^∗^	**0.91**	-0.11	0.20
Left dorsal posterior cingulate cortex^∗^	**0.84**	0.50	-0.10
Right ventral posterior cingulate cortex^∗^	**0.83**	-0.15	0.09
Left ventral posterior cingulate cortex^∗^	**0.83**	0.10	0.09
Left amygdala^∗^	**0.79**	0.18	-0.23
LH_aSPC	**0.66**	-0.12	**0.33**
RH_SFS	0.05	**0.86**	0.21
LH_SFS	-0.20	**0.83**	0.17
RH_vACC	0.11	**0.52**	-0.42
LH_STG	0.13	0.24	**0.68**
*Eigenvalue*	4.96	1.64	1.19
*% of variance*	49.61	16.4	11.91

*Loadings above the cut-off threshold are indicated in bold. Regions uniquely comprised in the first factor (but not in the other factors) are indicated by an asterisk*.

Subsequently, SEM was performed based on a Path Analysis Model with only observed variables (see for examples of a similar approach in neuroimaging research McIntosh, 1998; Horwitz et al., 1999; Ingvar and Petersson, 2000; Addis et al., 2007) by using the LISREL 8.7 statistical package (Joreskog and Sorbom, 2006). Path analysis allows to solve a set of simultaneous regression equations that theoretically establish the relationship among multiple variables (i.e., regional activation patterns) in a specified model (Anderson and Gerbing, 1988; MacCallum and Austin, 2000). Each ROI in the model defines a regression equation relating its pattern of neural response to the responses in the ROIs connected to it. The simultaneous system of equations is solved via least squares or maximum likelihood for the strengths of the interactions (the path coefficients) joining the regions. The standardized path coefficients can be interpreted as partial correlation or regression coefficients that convey assumptions about the directionality of ROI interactions for task performance.

Based on the literature on anatomical as well as functional connections between amygdala and FFG, and between FFG and PCC (but not between amygdala and PCC; Freese and Amaral, 2006; Vogt et al., 2006; Hagmann et al., 2008; Adolphs, 2010; Pessoa and Adolphs, 2010; Bzdok et al., 2013; Bickart et al., 2014), we tested four competitive models: (1) [PCC → left FFG → left amygdala]; (2) [amygdala → FFG → PCC]; (3) [PCC → FFG ↔ amygdala]; (4) [PCC ↔ FFG ← amygdala]. Thus, in specifying the models it was considered that the direction of functional interactions between the regions remains to be explored. The models can be considered equally complex, because characterized by the same number of parameters, while only differing in the directionality of the connections. Moreover, neither specific constraints were applied on the models nor parameters were released. It was not possible to test other possibilities of interactions between these regions due to the absence of an independent variable in those cases.

We tested these four models concerning the effects on BOLD response (i.e., average differential beta values from participants) due to stimulus congruence (congruent *versus* incongruent). As a further control analyses, the same models were also tested for the effects due to facial expression (pain *versus* pleasure) and tactile sensation (hit *versus* caress).

### Task-Free fMRI Data Preprocessing and Analysis

To investigate whether differential brain responses to congruent and incongruent stimuli (tactile sensation × facial expression interaction effect) could be explained by brain intrinsic functional organization, the relationship was tested between task-evoked neural responses (differences between beta values of the congruent and the incongruent conditions) in left amygdala, representing a final processing stage according to the SEM results, and intrinsic functional connectivity during task-free fMRI scanning with the other ROIs composing the model, representing previous processing stages. Intrinsic functional connectivity is operationally defined as the statistical dependence between low-frequency (0.009–0.08 Hz), task-independent BOLD fluctuations in distant brain regions and is considered to represent an index of intrinsic long-range communication across the brain (Van Dijk et al., 2010).

For intrinsic functional connectivity analysis of the task-free fMRI sessions, in addition to the fMRI preprocessing steps described for task-fMRI data, a second step of data preprocessing (Ebisch et al., 2011; Power et al., 2014) was performed on the task-free fMRI time series by using self-devised MATLAB (The Mathworks, Inc., Natick, MA) scripts. These included: (1) bandpass filtering between 0.009 and 0.08 Hz; (2) regression of global, white matter, and ventricle signals, and their first derivatives; (3) regression of three dimensional motion parameters, and their first derivatives; (4) scrubbing of motion affected functional volumes including frame-wise displacement (FD; threshold = 0.5%) and differential spatial variance (DVARS; threshold = 4.6%).

Since intrinsic functional connectivity analysis was performed on a separate task-free data set, more general and independent ROIs were created, that is, a priory voxel clusters defined as spheres with a 6 mm radius and functionally based on the peak coordinates of the activation clusters (showing a tactile sensation × facial expression interaction effect) included in the SEM analysis.

Connectivity indices were calculated (and transformed by Fisher r-to-z transformation) for each subject by correlating the average ROI time-courses from left amygdala with the average ROI time-courses from FFG, dorsal PCC and bilateral ventral PCC. Both individual task-evoked neural responses in amygdala and functional connectivity indices (z-scores based on the correlations) were transformed in natural log values in order to account for non-linear relationships. Finally, Pearson correlation coefficients were calculated between task-evoked neural responses in left amygdala and its functional connectivity indices during task-free fMRI with the other ROIs of the network (FFG, left/right vPCC, left dPCC).

## Results

### Behavioral Results of the fMRI Experiment

Analysis of task performance during the fMRI experiment showed that participants made on average 1.5 errors (standard deviation = 1.7; range: 0–5) when responding to the correct/incorrect questions throughout the experiment corresponding to an error rate of 3%. This suggests that the task was easy, that agreement among participants about stimulus congruence was high, and that participants attentively watched the relevant aspects of video content.

### Task fMRI Results: Stimulus Congruence

**FIGURE 2:**
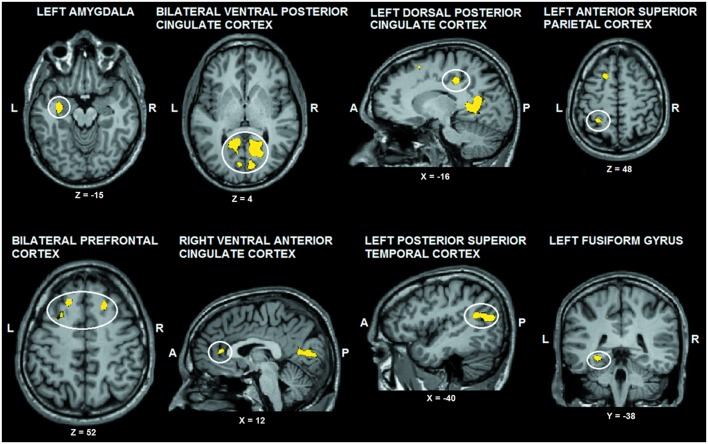
**Voxel clusters (thresholded at *p* < 0.05 corrected; *k* > 10) characterized by a significant interaction effect “facial expression × tactile sensation.”** BOLD responses in these clusters were stronger during the congruent conditions, compared to the incongruent conditions. L: left; R: right; A: anterior; P: posterior.

The tactile sensation × facial expression interaction [(caress-pleasure + hit-pain) *versus* (caress-pain + hit-pleasure)] was of principal interest for the study, because it indicates an effect of stimulus congruency. This statistical interaction based on ANOVA yielded significant clusters (*F*_1,25_ > 13.88; *p* < 0.001) in bilateral ventral PCC (vPCC), superior/lateral prefrontal cortex, left (ventrolateral) amygdala, dorsal PCC (dPCC), posterior superior temporal gyrus, anterior superior parietal cortex (aSPC) and anterior FFG, and right ventral anterior cingulate cortex (**Table 2**; **Figure 2**).

**Table 2:** Statistical and anatomical details about the voxel clusters characterized by a significant tactile sensation × facial expression interaction effect.

Brain region	Talairach coordinates (x/y/z) peak	Peak *F*-value	Uncorrected *p*-value of peak voxel	Cluster size	Experimental condition	Peak ß-value (± standard error)
Right PFC (BA8)	20/25/48	25.15	<0.00005	378	Caress-pleasure	-0.02 (± 0.04)
					Hit-pain	-0.05 (± 0.05)
					Caress-pain	-0.18 (± 0.04)
					Hit-pleasure	-0.11 (± 0.05)
Left PFC (BA8)	-16/13/54	28.22	<0.00005	1242	Caress-pleasure	-0.03 (± 0.03)
					Hit-pain	0.01 (± 0.03)
					Caress-pain	-0.11 (± 0.03)
					Hit-pleasure	-0.10 (± 0.02)
Left aSPC (BA 7)	-28/-44/48	24.37	<0.00005	243	Caress-pleasure	0.54 (± 0.08)
					Hit-pain	0.63 (± 0.07)
					Caress-pain	0.50 (± 0.08)
					Hit-pleasure	0.52 (± 0.07)
Left dPCC	-16/-32/39	27.96	<0.00005	837	Caress-pleasure	0.06 (± 0.03)
(BA 31)					Hit-pain	0.10 (± 0.03)
					Caress-pain	-0.03 (± 0.03)
					Hit-pleasure	0.00 (± 0.03)
Left vPCC	-7/-50/3	24.92	<0.00005	7749	Caress-pleasure	0.43 (± 0.15)
(BA 17/18/19/23/30)					Hit-pain	0.38 (± 0.15)
					Caress-pain	0.24 (± 0.15)
					Hit-pleasure	0.23 (± 0.14)
Right vPCC	11/-62/6	31.03	<0.00001	4860	Caress-pleasure	1.12 (± 0.19)
(BA 17/18/19/23/30)					Hit-pain	1.10 (± 0.18)
					Caress-pain	1.03 (± 0.18)
					Hit-pleasure	0.92 (± 0.18)
Right vACC	2/34/12	19.64	<0.0005	351	Caress-pleasure	-0.23 (± 0.05)
(BA 24/32)					Hit-pain	-0.18 (± 0.05)
					Caress-pain	-0.29 (± 0.05)
					Hit-pleasure	-0.40 (± 0.05)
Left amygdala	-34/-8/-15	39.13	<0.000005	837	Caress-pleasure	0.12 (± 0.03)
					Hit-pain	0.14 (± 0.02)
					Caress-pain	0.05 (± 0.03)
					Hit-pleasure	0.02 (± 0.02)
Left FFG	-28/-38/-9	19.00	<0.0005	405	Caress-pleasure	0.04 (± 0.04)
(BA 36/37)					Hit-pain	-0.03 (± 0.04)
					Caress-pain	-0.06 (± 0.05)
					Hit-pleasure	-0.12 (± 0.04)
Left STG	-40/-53/18	26.58	<0.00005	1755	Caress-pleasure	0.13 (± 0.06)
(BA 19/39)					Hit-pain	0.18 (± 0.07)
					Caress-pain	0.04 (± 0.07)
					Hit-pleasure	0.09 (± 0.07)

*BA, Brodmann area; PFC, prefrontal cortex; aSPC, anterior superior parietal cortex; d/vPCC, dorsal/ventral posterior cingulate cortex; vACC, ventral anterior cingulate cortex; FFG, fusiform gyrus; STG, superior temporal gyrus*.

**FIGURE 3:**
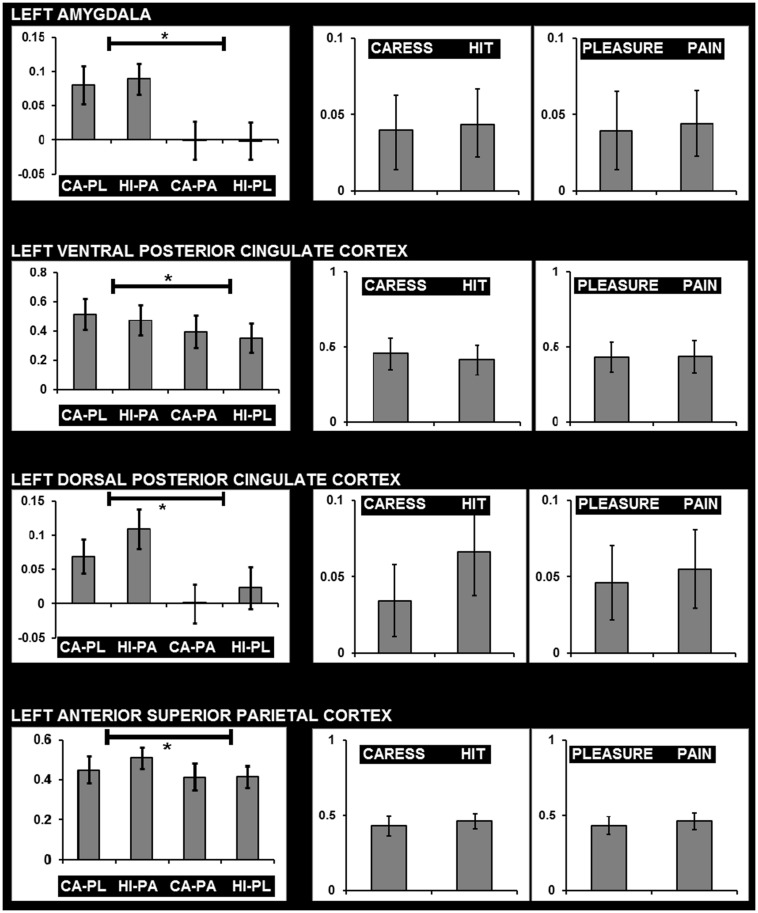
**Graphs showing average beta values and standard errors for the experimental conditions regarding voxels clusters characterized by a significant facial expression × tactile sensation interaction effect, *and* the absence of significant effects due tactile sensation or facial expression separately**. Graphs on the left show beta values for the four conditions (CA-PL: caress-pleasure; HI-PA: hit-pain; CA-PA: caress-pain; HI-PL: hit-pleasure) indicating stronger responses for congruent compared to incongruent stimuli. Graphs on the right show average beta values for caress *versus* hit stimuli, and pleasure *versus* pain stimuli, illustrating the absence of differences between the observation of distinct tactile sensations or distinct facial expressions in these regions. ^∗^, statistically significant difference *p* < 0.05.

For *post hoc* analysis, for each participant the beta values of the clusters obtained by the interaction contrast were calculated from the average signal time course of the voxels included in each cluster. Average beta values showed that activation in all these interaction ROIs was stronger during the congruent conditions, compared to the incongruent conditions (**Table 2**; **Figure 3**). No brain regions were characterized by increased activity for the incongruent conditions, compared to the congruent conditions, even when using a threshold of *p* < 0.01 uncorrected.

An additional control analysis using paired-sample *t*-tests was performed to rule out the possibility that increased BOLD responses to the congruent stimuli could be attributed to a cumulative processing of information from different sources regarding specific emotional content (e.g., expression and sensation of pain or expression and sensation of pleasure). The brain regions that were exclusively characterized by an interaction effect (congruent > incongruent) in the absence of a tactile sensation or facial expression effect (neither significant nor trend; *p* > 0.1) were left (ventrolateral) amygdala, left dPCC, left vPCC, and left aSPC (**Figure 3**).

### Task fMRI Results: Main Effects of Tactile Sensation and Facial Expression

**FIGURE 4:**
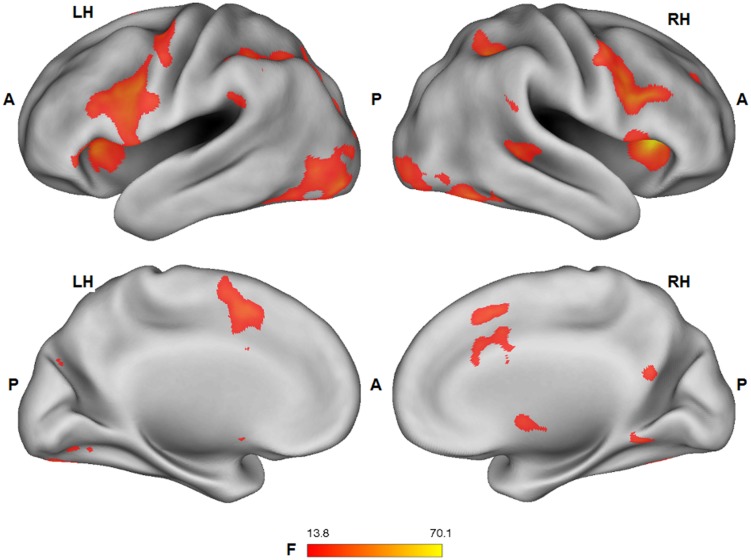
**Group statistical maps (*F*-statistics) showing voxels characterized by a significant effect of the facial expression factor (thresholded at *p* < 0.05 corrected; *k* > 10)**. L: left; R: right; A: anterior; P: posterior.

Group statistical fMRI maps (ANOVA: *F*_1,25_ > 13.88; *p* < 0.001) showed a significant main effect of the facial expression factor in bilateral dorsal anterior cingulate/supplementary motor cortex, ventral and dorsal premotor cortex, lateral prefrontal cortex, inferior frontal gyrus, anterior insula, nucleus caudatus, inferior parietal lobule/supramarginal gyrus, extrastriate cortex, fusiform gyrus, inferior temporal cortex, PCC, and right superior temporal sulcus. Except for PCC (pleasure > pain), BOLD response in these regions was stronger for the expression of pain, compared to the expression of pleasure (**Figure 4**).

**FIGURE 5:**
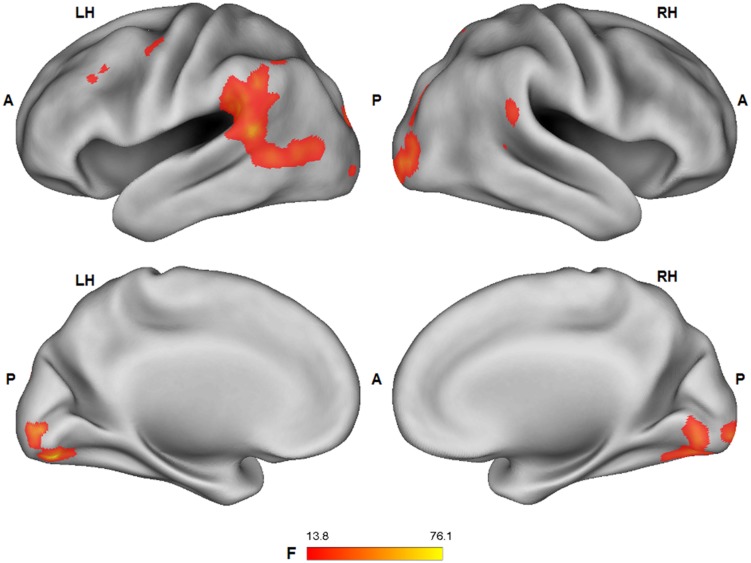
**Group statistical maps (*F*-statistics) showing voxels characterized by a significant effect of the tactile sensation expression factor (thresholded at *p* < 0.05 corrected; *k* > 10)**. L: left; R: right; A: anterior; P: posterior.

A significant main effect (ANOVA: *F*_1,25_ > 13.88; *p* < 0.001) of the tactile sensation factor was detected in bilateral lateral post-central gyrus (caress > hit), anterior temporal-parietal junction (hit > caress), left dorsal precentral gyrus (hit > caress), inferior parietal lobule/supramarginal gyrus (hit > caress), superior temporal gyrus (hit > caress), posterior parietal cortex (caress > hit), left occipital cortex/fusiform gyrus, and right occipital cortex/fusiform gyrus (caress > hit; **Figure 5**).

### Task fMRI Results: Overlap between Main Effects of Tactile Sensation and Facial Expression

**FIGURE 6:**
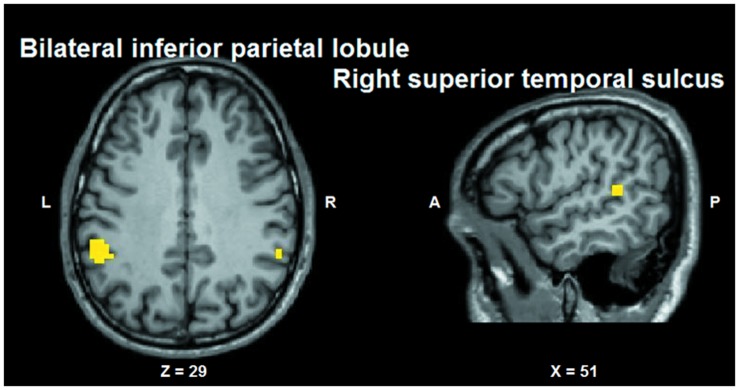
**Group statistical maps (conjunction thresholded at *p* < 0.05 corrected; *k* > 10) showing overlapping modulation by the observation of different tactile sensations as well as by different facial expressions**. L: left; R: right; A: anterior; P: posterior.

Conjunction analysis was performed to test whether voxel clusters existed that were modulated both by the tactile sensation factor and by the facial expression factor. Such a characteristic could provide a neural substrate allowing the convergence of these types of information. Conjunction analysis was based on the minimum statistic for the conjunction null (Nichols et al., 2005) and concerned the contrast [(caress-pleasure + caress-pain) *versus* (hit-pleasure + hit-pain)] ∩ [(caress-pleasure + hit-pleasure) *versus* (caress-pain + hit-pain)]. This analysis yielded overlapping voxel clusters (*t*_25_ > 3.72; *p* < 0.001) for the tactile sensation and the facial expression factors in bilateral anterior inferior parietal lobule (aIPL)/supramarginal gyrus and right superior temporal gyrus (STS; **Figure 6**).

### Task fMRI Results: SEM

Regarding SEM, the fit of each model was assessed by means of the following goodness of fit indices: (1) the chi-square (χ^2^) statistic and its degrees of freedom; (2) the root mean square error of approximation (RMSEA) and its 90% confidence interval (90% CI); (3) the Non-Normed Fit Index (NNFI), 4) the Comparative Fit Index (CFI); and (5) the standardized root mean square residuals (SRMRs). Furthermore, to compare the alternative models, the Expected Cross-Validation Index (ECVI) was used. A model was considered to the data when: χ^2^/df ≤ 2, CFI and NNFI ≥ 0.97, SRMR ≤ 0.05, and RMSEA ≤ 0.05 (90% CI: the lower boundary of the CI should contain zero for exact fit and be <0.05 for close fit). A model with an ECVI smaller than the ECVI-for-comparison model should be preferred. Based on the fit values, the models can be categorized in (1) under/not-identified models if one or more parameters may not be uniquely determined, because there is not enough information; (2) just-identified models when all of the parameters are uniquely determined, and (3) over-identified models if there is more than one way of estimating a parameter and therefore are not exhaustive (Hooper et al., 2008; Kline, 2015). Thus, a just-identified model provides the best solution to describe the data.

**FIGURE 7:**
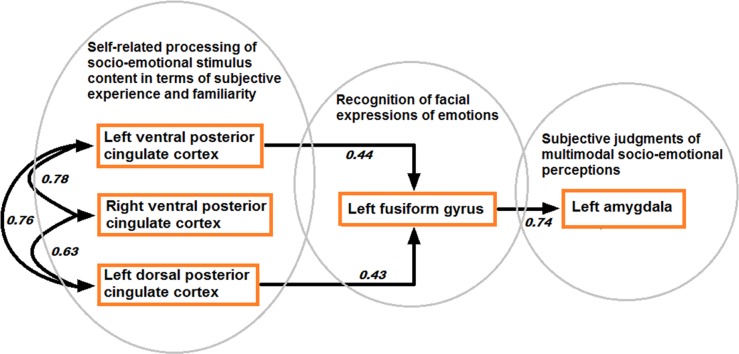
**Model of directional effects (indicating standardized path coefficients) between posterior cingulate cortex, fusiform gyrus, and amygdala obtained by SEM for the differentiation between congruent and incongruent stimuli, and a recapitulation of the task-related fMRI results concerning the encoding of socio-emotional stimulus congruence within the network**.

Structural equation modeling confirmed the model based on directional effects from left vPCC and dPCC to left fusiform gyrus, and from left fusiform gyrus to left amygdala, while strong bidirectional interactions were found between bilateral vPCC and left dPCC (model 1 visualized in **Figure 7**). The other models were characterized by an over-fit (model 3; not satisfying the goal of parsimony by adding unnecessary parameters resulting in a too complex model which is impossible to falsify) or yielded inadequate fit indices (models 2 and 4). Moreover, when testing the models in the control contexts of effects due to tactile sensations and facial expressions, inadequate fit indices were obtained in all cases. Statistical data of the different models in the different contexts are presented in **Table 3**.

**Table 3:** Structural equation modeling (SEM) fit values for the four alternative models applied to the three experimental factors (tactile sensation, facial expression, tactile sensation × facial expression).

Factors/models	χ^2^	df	χ^2^/df	NNFI	CFI	RMSEA	90% CI	SRMR	ECVI
***Expression × sensation (congruence)***									
Model 1^∗∗^	3.69	4	0.92	0.99	0.99	0.00	0.00-0.31	0.06	1.41
Model 2	12.87	5	2.57	0.80	0.90	0.26	0.08-0.83	0.35	179
Model 3	0.66	3	0.22	1.07	1.00	0.00	0.00-0.17	0.02	1.36
Model 4	15.55	3	5.18	0.64	0.89	0.40	0.21–0.61	0.140	2.02
***Expression***									
Model 1	14.15	4	3.54	0.65	0.86	0.34	0.10–1.16	0.11	2.10
Model 2	21.00	5	4.2	0.41	0.70	0.37	0.21–0.53	0.35	2.12
Model 3	11.83	3	3.94	0.53	0.86	0.37	0.16-0.70	0.12	2.08
Model 4	4.54	3	1.51	0.93	0.98	0.15	0.00-0.40	0.073	1.61
***Sensation***									
Model 1	4.97	4	1.24	0.94	0.98	0.10	0.00–0.35	0.06	1.68
Model 2	11.77	5	2.35	0.65	0.82	0.25	0.01–0.87	0.25	1.74
Model 3	4.93	3	1.64	0.87	0.96	0.17	0.00–0.56	0.06	1.77
Model 4	13.01	3	4.34	0.22	0.77	0.37	0.18–0.59	0.160	1.96

*NNFI, Non-Normed Fit index; CFI, Comparative Fit Index; RMSEA, root mean square error of approximation; 90% CI, RMSEA 90% confidence interval; SRMR, standardized root mean square residual; ECVI, Expected Cross-Validation Index; ^∗∗^ model characterized by adequate fit indices*.

*Model 1: PCC → FFG → amygdala;*

*Model 2: PCC → FFG ↔ amygdala;*

*Model 3: amygdala → FFG → PCC;*

*Model 4: PCC ↔ FFG ← amygdala*.

### Task-Free fMRI Results: Intrinsic Functional Connectivity

**FIGURE 8:**
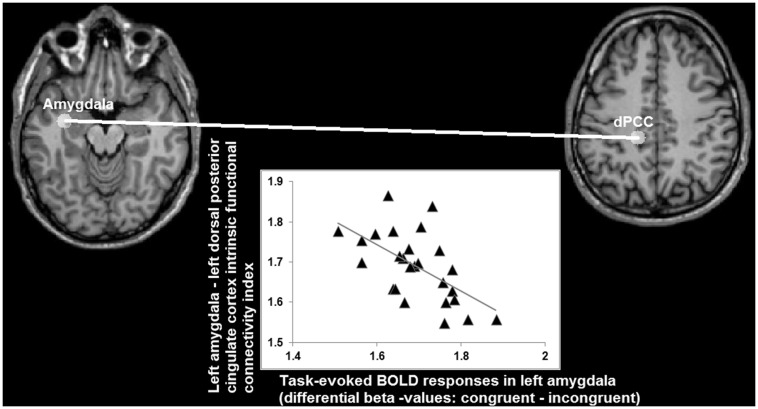
**Relationship (*r* = -0.58) between differential BOLD responses in left amygdala for congruent and incongruent stimuli, and its intrinsic functional connectivity with left dorsal posterior cingulate cortex (axes indicating natural log values)**.

Analysis of the statistical dependency between differential beta values in left amygdala (congruent *minus* incongruent; obtained from the task-fMRI data set) and functional connectivity indices (obtained from the task-free fMRI data set) yielded a significant correlation (*r* = -0.58; *p* < 0.008 Bonferroni corrected for multiple comparisons) between the congruency effects in left amygdala during task-performance and its connectivity with left dPCC, but not with the others ROIs (FFG: *r* = -0.05; left vPCC: -0.08; right vPCC: -0.17, all *p* > 0.5 Bonferroni corrected for multiple comparisons), during the task-free fMRI blocks (**Figure 8**).

When looking at the intrinsic functional connectivity indices during the task-free state at the group level, an average correlation between BOLD low-frequency fluctuations in left amygdala and left dPCC of *r* = -0.01 is detected (*SD* = 0.29; minimum *r* = -0.42; maximum *r* = 0.67; *t* = -0.0617; *p* = 0.5). Therefore, these data imply that a stronger differentiation between the congruent and incongruent stimuli is accompanied by an absence or even antagonistic relationship (i.e., a negative correlation coefficient) between intrinsic activity in left amygdala and left dPCC. Vice versa, the more left amygdala and dPCC co-vary during a task-free state, the lesser the differentiation between congruent and incongruent stimuli in amygdala during task-performance.

## Discussion

This study aimed at investigating the integrative processing of information from multiple sources during social perception, in particular others’ tactile sensations and facial expressions of emotion. Integrative processing as represented by an effect of stimulus congruence was detected in left ventrolateral amygdala, left dPCC, left vPCC, and left aSPC. In all cases, neural activity was stronger when the facial expression was congruent with the tactile sensation regarding valence. Furthermore, no additional effects due to tactile sensation or facial expression was observed in these brain regions, that is, the congruency effects did not differ as a function of emotion or sensation. These findings show that congruent, unimodal social stimuli with emotional content can naturally induce stronger responses of affective networks, especially amygdala and PCC.

Amygdala has been proposed as a key structure in the human brain facilitating social life (Adolphs, 2010; Bickart et al., 2014). Its functions are strongly related to encoding emotional relevance as well as salience or impact of stimuli (Ewbank et al., 2009; Pessoa and Adolphs, 2010) independent of stimulus valence (Fitzgerald et al., 2006; van der Gaag et al., 2007). Particularly relevant for social perception, amygdala supports the recognition of facial expressions of emotion (Adolphs, 2002) and amygdala neurons were found to encode subjective judgments of emotional faces (Wang et al., 2014). In addition, the location of the amygdala cluster in the present study corresponds most closely to the ventrolateral amygdala (Ball et al., 2009; Saygin et al., 2011; Bzdok et al., 2013) embedded in a network supporting the integration of multisensory information from the environment with self-relevant cognition (Adolphs, 2010; Saygin et al., 2011; Bickart et al., 2014) and awareness of others’ emotions (Bickart et al., 2014).

A parsimonious interpretation of BOLD modulations due to stimulus congruence in ventrolateral amygdala would thus be that they reflect an augmented self-relevance or impact of congruent socio-emotional stimuli. Alternatively, facial expressions of emotion that correspond to what one would expect from the context in which they occur, would facilitate subjective judgments of the depicted emotional experiences in amygdala. Such expectations could be based on one’s personal experiences with a certain situation. For instance, when witnessing someone expressing pain while being hit by someone, it is likely easier to judge the other’s affective experience as one is familiar with the experience of expressing pain while being hit. The latter interpretation seems to be favored when considering the full picture of results.

Several other brain regions showed activation patterns similar to those detected in amygdala, including FFG and v/dPCC. FFG is a main structure sending afferent information to amygdala with a principal role in the perception of faces and emotional expressions (Kanwisher et al., 1997; McCarthy et al., 1997; Fairhall and Ishai, 2007; Herrington et al., 2011; Saygin et al., 2012). PCC, instead, contributes to self-reflection and the integration of self-referential stimuli in one’s own personal context (Northoff and Bermpohl, 2004; van der Meer et al., 2010), functions that can be extended to social cognition, too (Schilbach et al., 2008). These functions are also supported by the involvement of PCC in autobiographical memory (Svoboda et al., 2006; Spreng and Grady, 2010) and in the interaction between memory and emotion (Maddock et al., 2003; van der Meer et al., 2010).

To investigate the mutual relationships among amygdala, FFG and PCC, SEM was performed on the fMRI data as an exploratory analysis. SEM results preliminary confirmed a model presuming that amygdala activity depended on earlier processing stages in left FFG, whereas individual FFG activity in turn could be predicted by activity patterns in left vPCC and dPCC. Left vPCC and dPCC also interacted with each other and right vPCC. Although, some studies suggested early processing of emotional faces in amygdala (Vuilleumier et al., 2004; Vuilleumier and Pourtois, 2007) influencing FFG activity, others showed amygdala activity being mediated by visual and fusiform cortices (Haxby et al., 2002). Amygdala is possibly involved in multiple processing stages of complex socio-emotional stimuli (Adolphs et al., 2002; Pourtois et al., 2010).

The present results indicating a late, higher-level involvement of amygdala can be argued to be in line with the experimental context implying the explicit comprehension and integration of multimodal aspects of social stimuli, like emotional expressions and bodily sensations. In particular, the task may have put the participants in a cognitive perspective by requiring the explicit differentiation between congruent and incongruent stimuli. Accordingly, relatively long response latencies of amygdala neurons were also reported during the subjective, explicit recognition of facial expressions of emotion (Wang et al., 2014). Furthermore, whereas effects were found to be mainly left lateralized, socio-emotional functions of the amygdala, including the subjective recognition of emotion, may be bilateral (Baas et al., 2004; Mobbs et al., 2006; Wang et al., 2014). However, literature remains somewhat inconclusive about functional differences between left and right amygdala (Adolphs, 2010), and some models suggest that left amygdala more specifically contributes to cognitive perceptual processing of emotional information in contrast to more fast and automatic responses in right amygdala (Gläscher and Adolphs, 2003).

In addition to amygdala, PCC might add subjective information about self-relatedness or familiarity of socio-emotional stimuli to further stimulus encoding in FFG and amygdala (Northoff and Bermpohl, 2004; Schilbach et al., 2008). In support of a possible link between the coding of stimulus congruence and self-relatedness of stimulus content or stimulus familiarity, stimulus evaluations in an independent sample of participants showed that the congruent videos were perceived as significantly more self-related in terms of subjective experiences as well as more familiar based on personal past experiences.

One exception to these rating results was the *post hoc* contrasts between the hit-pain (congruent) and the hit-pleasure (incongruent) condition, given that no significant difference was detected concerning self-relatedness and familiarity. This possibly could be attributed to the fact that on average facial expressions of pleasure also were judged as being more self-related as well as more familiar than facial expressions of pain. Moreover, while the fMRI task (judging stimulus congruence) explicitly required participants to consider both the tactile sensation and the facial expression, this was not the case for the judgments of self-relatedness and familiarity. Thus, participants also may have focused more on the facial expression than the tactile sensation during the self-relatedness/familiarity judgments. Further experiments are warranted to disentangle these aspects.

However, because self-relatedness/familiarity judgments and fMRI data were obtained in different samples of participants, the link between self-relatedness/familiarity judgments and stimulus congruence coding remains speculative, and these rating results cannot be related directly to the fMRI data. Directly relating stimulus judgments and neural activity patterns would be necessary to test the hypothesized link between integrative processing of social stimulus content and self-processing in subsequent studies by correlating such variables within the same sample.

Notably, the results obtained by intrinsic functional connectivity analysis on a separate task-free dataset from the same participants further showed that differentiation between congruent and incongruent social stimuli in left amygdala inversely depended on its intrinsic functional connectivity with left dPCC: a weaker or more negative relationship between amygdala and dPCC during a task-free state (i.e., a state of intrinsic or spontaneous activity patterns without being involved in a specific task) was associated with an increased differentiation between congruent and incongruent social stimuli during task-performance. In line with previous studies (Roy et al., 2009; Leech et al., 2012), the absence of a significant positive functional connectivity as observed in the present study suggested that amygdala and PCC belong to distinct networks that are functionally independent when not performing a specific task. Whereas the relationship between task-evoked activity and intrinsic functional connectivity has been investigated by relatively few studies (e.g., Fox et al., 2006; Mennes et al., 2010; Touroutoglou et al., 2014), a negative relationship also has been reported by a previous study (He, 2013).

The relationship between task-evoked activity in amygdala and intrinsic functional connectivity between amygdala and dPCC might reflect neural predisposition explaining inter-individual variability in the integrative processing of social stimulus content (Northoff, 2013, 2014). An increased independence of spontaneous activity in amygdala from dPCC in an individual may allow amygdala to respond more dynamically to certain environmental stimuli. Relevantly, PCC has been identified as a central brain hub characterized by a topology that allows switching and integration of processing in different networks involved in internally and externally guided information processing (Leech et al., 2012; de Pasquale et al., 2015). Alterations in the interaction between internally (e.g., self-related processing) and externally guided (e.g., social stimuli) information processing may be especially relevant as a putative mechanism explaining certain psychopathological phenomena typically observed in psychosis, such as disturbances in self-other relationship (Ebisch and Aleman, 2016).

Some other issues need to be mentioned. As detected by a conjunction analysis investigating the overlap between the main effects of facial expression and tactile sensation, the only brain regions modulated by both factors were left IPL/supramarginal gyrus and right STS. The aIPL activation cluster likely is located in the putative human homolog of macaque area PF/PFG (Caspers et al., 2006), a multisensory region with motor, somatosensory, visual and mirror properties (Fogassi et al., 2005; Rozzi et al., 2008). In humans, it was found to be involved in action observation and imitation (Caspers et al., 2010), and the observation of others’ tactile experiences (Ebisch et al., 2008; Morrison et al., 2013). Based on this information, we propose that information about others’ tactile experiences can converge with the motor aspects of others’ facial expressions of emotion based on multimodal integration and mirror properties of aIPL.

Secondly, although both facial expressions and tactile sensations depicted affective experiences, no statistical interactions between these aspects were found in brain regions commonly implicated in the understanding of others’ affective experiences, like anterior insula, supplementary motor cortex, or orbitofrontal cortex (Bastiaansen et al., 2009; Fan et al., 2011; Bernhardt and Singer, 2012; Hillis, 2014; Lamm et al., 2015). However, a possible explanation is provided by an fMRI study (Di Dio et al., 2007) illustrating a subjective encoding of external stimuli in amygdala (i.e., beauty judgments of artworks), in contrast to objective processing in insula (i.e., watching canonical masterpieces, compared to modified versions of these pictures). Similarly, in the present study congruency of information provided by facial expressions and bodily experiences can be considered a mere subjective judgment associated with amygdala. Because in the present study a large consensus existed across participants regarding stimulus (in)congruence (>95%), this interpretation could not be tested directly. Further studies are warranted to elucidate the effects of stimulus congruence in interaction with inter-individual variability in previous subjective experiences of this congruence within the same participants (e.g., familiarity or personal relevance).

Finally, in the present study the incongruent stimuli did not induce any increase of neural activity, compared to the congruent stimuli. Incongruent stimuli could be expected to recruit mentalizing networks (de Lange et al., 2008; Van Overwalle and Baetens, 2009; Schurz et al., 2014), while higher cognitive demands can suppress affect processing (Okon-Singer et al., 2013), and incongruent conditions could lead to higher conflict processing (Etkin et al., 2006; Klasen et al., 2011; Muller et al., 2011). However, although the congruence judgment task likely provided an explicit context, it must be noted that, in contrast to previous studies, we did not require participants to make forced choices or decisions about the experiences of the actors in the videos in case of contradictory information. These characteristics of the paradigm kept cognitive demands and conflict processing minimal. Therefore, the present findings suggest that social situations that are less intuitive to understand (e.g., incongruent stimuli) are not automatically associated with higher demands on social reasoning or conflict processes, though it also must be mentioned that the experimental paradigm may not have excluded conflict processing completely.

## Conclusion

The present findings suggest that a network including PCC, FFG, and amygdala is involved in the integrative processing of social information from manifold bodily sources about others’ feelings. In particular, these results imply that the natural perception of coherent social situations has a higher socio-emotional impact or self relevance than ambiguous perceptions involving a network related to emotion and self-related processing. Directly investigating the hypothesized link between integrative processing during social perception and self-related processing within the same sample may represent an important topic for subsequent studies.

## Author Contributions

Study conception and design: SE, VG, Anatolia Salone, MG, GLR; acquisition of data and participant recruitment: SE, Anatolia Salone, GM, MP; data processing: SE, Anatolia Salone; data analysis: SE, DM, GN, LC, Aristide Saggino; writing the manuscript: SE, Anatolia Salone, GM, LC, DM, MP, Aristide Saggino, GLR, MG, GN, VG.

## Conflict of Interest Statement

The authors declare that the research was conducted in the absence of any commercial or financial relationships that could be construed as a potential conflict of interest.

## References

[B1] Addis D. R. Moscovitch M. McAndrews M. P. (2007). Consequences of hippocampal damage across the autobiographical memory network in left temporal lobe epilepsy. *Brain* 130 2327–2342. 10.1093/brain/awm166 17681983

[B2] Adolphs R. (2002). Recognizing emotion from facial expressions: psychological and neurological mechanisms. *Behav. Cogn. Neurosci. Rev.* 1 21–62. 10.1177/1534582302001001003 17715585

[B3] Adolphs R. (2010). What does the amygdala contribute to social cognition? *Ann. N. Y. Acad. Sci.* 1191 42–61. 10.1111/j.1749-6632.2010.05445.x 20392275 PMC2871162

[B4] Adolphs R. Baron-Cohen S. Tranel D. (2002). Impaired recognition of social emotions following amygdala damage. *J. Cogn. Neurosci.* 14 1264–1274. 10.1162/089892902760807258 12495531

[B5] Adolphs R. Damasio H. Tranel D. Cooper G. Damasio A. R. (2000). A role for somatosensory cortices in the visual recognition of emotion as revealed by three-dimensional lesion mapping. *J. Neurosci.* 20 2683–2690. 10729349 10.1523/JNEUROSCI.20-07-02683.2000 PMC6772225

[B6] Anderson J. C. Gerbing D. W. (1988). Structural equation modeling in practice: a review and recommended two-step approach. *Psychol. Bull.* 103 411–423. 10.1037/0033-2909.103.3.411

[B7] Baas D. Aleman A. Kahn R. S. (2004). Lateralization of amygdala activation: a systematic review of functional neuroimaging studies. *Brain Res.* 45 96–103. 10.1016/j.brainresrev.2004.02.004 15145620

[B8] Ball T. Derix J. Wentlandt J. Wieckhorst B. Speck O. Schulze-Bonhage A. (2009). Anatomical specificity of functional amygdala imaging of responses to stimuli with positive and negative emotional valence. *J. Neurosci. Methods* 180 57–70. 10.1016/j.jneumeth.2009.02.022 19427530

[B9] Bastiaansen J. A. Thioux M. Keysers C. (2009). Evidence for mirror systems in emotions. *Philos. Trans. R. Soc. B* 364 2391–2404. 10.1098/rstb.2009.0058 PMC2865077 19620110

[B10] Bernhardt B. C. Singer T. (2012). The neural basis of empathy. *Ann. Rev. Neurosci.* 35 1–23. 10.1146/annurev-neuro-062111-150536 22715878

[B11] Bickart K. C. Dickerson B. C. Barrett L. F. (2014). The amygdala as a hub in brain networks that support social life. *Neuropsychologia* 63 235–248. 10.1016/j.neuropsychologia.2014.08.013 25152530 PMC4981504

[B12] Bzdok D. Laird A. R. Zilles K. Fox P. T. Eickhoff S. B. (2013). An investigation of the structural, connectional, and functional subspecialization in the human amygdala. *Hum. Brain Mapp.* 34 3247–3266. 10.1002/hbm.22138 22806915 PMC4801486

[B13] Caspers S. Geyer S. Schleicher A. Mohlberg H. Amunts K. Zilles K. (2006). The human inferior parietal cortex: cytoarchitectonic parcellation and interindividual variability. *Neuroimage* 33 430–448. 10.1016/j.neuroimage.2006.06.054 16949304

[B14] Caspers S. Zilles K. Laird A. R. Eickhoff S. B. (2010). ALE meta-analysis of action observation and imitation in the human brain. *Neuroimage* 50 1148–1167. 10.1016/j.neuroimage.2009.12.112 20056149 PMC4981639

[B15] Cattell R. B. (1966). The scree test for the number of factors. *Multivar. Behav. Res.* 1 245–276. 10.1207/s15327906mbr0102_10 26828106

[B16] Cox R. W. (1996). AFNI: software for analysis and visualization of functional magnetic resonance neuroimages. *Comput. Biomed. Res.* 29 162–173. 10.1006/cbmr.1996.0014 8812068

[B17] Craig A. D. (2009). How do you feel–now? The anterior insula and human awareness. *Nat. Rev. Neurosci.* 10 59–70. 10.1038/nrn2555 19096369

[B18] de Lange F. P. Spronk M. Willems R. M. Toni I. Bekkering H. (2008). Complementary systems for understanding action intentions. *Curr. Biol.* 18 454–457. 10.1016/j.cub.2008.02.057 18356050

[B19] de Pasquale F. Della Penna S. Sporns O. Romani G. L. Corbetta M. (2015). A dynamic core network and global efficiency in the resting human brain. *Cereb. Cortex* 10.1093/cercor/bhv185 PMC5027996 26347485

[B20] Di Dio C. Macaluso E. Rizzolatti G. (2007). The golden beauty: brain response to classical and renaissance sculptures. *PLoS ONE* 2:e1201 10.1371/journal.pone.0001201 PMC2065898 18030335

[B21] Ebisch S. J. Aleman A. (2016). The fragmented self: imbalance between intrinsic, and extrinsic self-networks in psychotic disorders. *Lancet Psychiatry* 10.1016/S2215-0366(16)00045-6 27374147

[B22] Ebisch S. J. Gallese V. Willems R. M. Mantini D. Groen W. B. Romani G. L. (2011). Altered intrinsic functional connectivity of anterior and posterior insula regions in high-functioning participants with autism spectrum disorder. *Hum. Brain Mapp.* 32 1013–1028. 10.1002/hbm.21085 20645311 PMC6870194

[B23] Ebisch S. J. Perrucci M. G. Ferretti A. Del Gratta C. Romani G. L. Gallese V. (2008). The sense of touch: embodied simulation in a visuotactile mirroring mechanism for observed animate or inanimate touch. *J. Cogn. Neurosci.* 20 1611–1623. 10.1162/jocn.2008.20111 18345991

[B24] Etkin A. Egner T. Peraza D. M. Kandel E. R. Hirsch J. (2006). Resolving emotional conflict: a role for the rostral anterior cingulate cortex in modulating activity in the amygdala. *Neuron* 51 871–882. 10.1016/j.neuron.2006.07.029 16982430

[B25] Ewbank M. P. Barnard P. J. Croucher C. J. Ramponi C. Calder A. J. (2009). The amygdala response to images with impact. *Soc. Cogn. Affect. Neurosci.* 4 127–133. 10.1093/scan/nsn048 19151376 PMC2686226

[B26] Fairhall S. L. Ishai A. (2007). Effective connectivity within the distributed cortical network for face perception. *Cereb. Cortex* 17 2400–2406. 10.1093/cercor/bhl148 17190969

[B27] Fan Y. Duncan N. W. de Greck M. Northoff G. (2011). Is there a core neural network in empathy? An fMRI based quantitative meta-analysis. *Neurosci. Biobehav. Rev.* 35 903–911. 10.1016/j.neubiorev.2010.10.009 20974173

[B28] Fitzgerald D. A. Angstadt M. Jelsone L. M. Nathan P. J. Phan K. L. (2006). Beyond threat: amygdala reactivity across multiple expressions of facial affect. *Neuroimage* 30 1441–1448. 10.1016/j.neuroimage.2005.11.003 16368249

[B29] Fogassi L. Ferrari P. F. Gesierich B. Rozzi S. Chersi F. Rizzolatti G. (2005). Parietal lobe: from action organization to intention understanding. *Science* 308 662–667. 10.1126/science.1106138 15860620

[B30] Forman S. D. Cohen J. D. Fitzgerald M. Eddy W. F. Mintun M. A. Noll D. C. (1995). Improved assessment of significant activation in functional magnetic resonance imaging (fMRI): use of a cluster-size threshold. *Magn. Reson. Med.* 33 636–647. 10.1002/mrm.1910330508 7596267

[B31] Fox M. D. Snyder A. Z. Zacks J. M. Raichle M. E. (2006). Coherent spontaneous activity accounts for trial-to-trial variability in human evoked brain responses. *Nat. Neurosci.* 9 23–25. 10.1038/nn1616 16341210

[B32] Freese J. L. Amaral D. G. (2006). Synaptic organization of projections from the amygdala to visual cortical areas TE and V1 in the macaque monkey. *J. Comput. Neurol.* 496 655–667. 10.1002/cne.20945 PMC2564872 16615120

[B33] Friston K. J. Buechel C. Fink G. R. Morris J. Rolls E. Dolan R. J. (1997). Psychophysiological and modulatory interactions in neuroimaging. *Neuroimage* 6 218–229. 10.1006/nimg.1997.0291 9344826

[B34] Gallese V. (2003). The roots of empathy: the shared manifold hypothesis and the neural basis of intersubjectivity. *Psychopathology* 36 171–180. 10.1159/000072786 14504450

[B35] Gallese V. Ebisch S. (2013). Embodied simulation and touch: the sense of touch in social cognition. *Phenomenol. Mind.* 4 269–291.

[B36] Gallese V. Keysers C. Rizzolatti G. (2004). A unifying view of the basis of social cognition. *Trends Cogn. Sci.* 8 396–403. 10.1016/j.tics.2004.07.002 15350240

[B37] Gläscher J. Adolphs R. (2003). Processing of the arousal of subliminal and supraliminal emotional stimuli by the human amygdala. *J. Neurosci.* 23 10274–10282. 14614086 10.1523/JNEUROSCI.23-32-10274.2003 PMC6741000

[B38] Hagmann P. Cammoun L. Gigandet X. Meuli R. Honey C. J. Wedeen V. J. (2008). Mapping the structural core of human cerebral cortex. *PLoS Biol.* 6:e159 10.1371/journal.pbio.0060159 PMC2443193 18597554

[B39] Handwerker D. A. Gonzalez-Castillo J. D’Esposito M. Bandettini P. A. (2012). The continuing challenge of understanding and modeling hemodynamic variation in fMRI. *Neuroimage* 62 1017–1023. 10.1016/j.neuroimage.2012.02.015 22366081 PMC4180210

[B40] Haxby J. V. Hoffman E. A. Gobbini M. I. (2002). Human neural systems for face recognition and social communication. *Biol. Psychiatry* 51 59–67. 10.1016/S0006-3223(01)01330-0 11801231

[B41] He B. J. (2013). Spontaneous and task-evoked brain activity negatively interact. *J. Neurosci.* 33 4672–4682. 10.1523/JNEUROSCI.2922-12.2013 23486941 PMC3637953

[B42] Herrington J. D. Taylor J. M. Grupe D. W. Curby K. M. Schultz R. T. (2011). Bidirectional communication between amygdala and fusiform gyrus during facial recognition. *Neuroimage* 56 2348–2355. 10.1016/j.neuroimage.2011.03.072 21497657 PMC3137553

[B43] Hillis A. E. (2014). Inability to empathize: brain lesions that disrupt sharing and understanding another’s emotions. *Brain* 137 981–997. 10.1093/brain/awt317 24293265 PMC3959550

[B44] Hooper D. Coughlan J. Mullen M. R. (2008). Structural equation modeling: guidelines for determining model fit. *Electron. J. Bus. Res. Methods* 6 53–60.

[B45] Horwitz B. Tagamets M. A. McIntosh A. R. (1999). Neural modeling, functional brain imaging, and cognition. *Trends Cogn. Sci.* 3 91–98. 10.1016/S1364-6613(99)01282-6 10322460

[B46] Ingvar M. Petersson K. M. (2000). *Functional Maps and Brain Networks, Brain Mapping: The Systems.* (Cambridge: Academic Press), 111–140.

[B47] Joreskog K. G. Sorbom D. (2006). *LISREL for Windows.* Lincolnwood, IL: Scientific Software International.

[B48] Kanwisher N. McDermott J. Chun M. M. (1997). The fusiform face area: a module in human extrastriate cortex specialized for face perception. *J. Neurosci.* 17 4302–4311. 9151747 10.1523/JNEUROSCI.17-11-04302.1997 PMC6573547

[B49] Keysers C. Kaas J. H. Gazzola V. (2010). Somatosensation in social perception. *Nat. Rev. Neurosci.* 11 417–428. 10.1038/nrn2833 20445542

[B50] Klasen M. Kenworthy C. A. Mathiak K. A. Kircher T. T. Mathiak K. (2011). Supramodal representation of emotions. *J. Neurosci.* 31 13635–13643. 10.1523/JNEUROSCI.2833-11.2011 21940454 PMC6623280

[B51] Kline P. (1994). *An Easy Guide to Factor Analysis.* London: Routledge.

[B52] Kline R. B. (2015). *Principles and Practice of Structural Equation Modeling.* New York City: Guilford publications.

[B53] Lamm C. Silani G. Singer T. (2015). Distinct neural networks underlying empathy for pleasant and unpleasant touch. *Cortex* 70 79–89. 10.1016/j.cortex.2015.01.021 25725510

[B54] Leech R. Braga R. Sharp D. J. (2012). Echoes of the brain within the posterior cingulate cortex. *J. Neurosci.* 32 215–222. 10.1523/JNEUROSCI.3689-11.2012 22219283 PMC6621313

[B55] MacCallum R. C. Austin J. T. (2000). Applications of structural equation modeling in psychological research. *Ann. Rev. Psychol.* 51 201–226. 10.1146/annurev.psych.51.1.201 10751970

[B56] Maddock R. J. Garrett A. S. Buonocore M. H. (2003). Posterior cingulate cortex activation by emotional words: fMRI evidence from a valence decision task. *Hum. Brain Mapp.* 18 30–41. 10.1002/hbm.10075 12454910 PMC6871991

[B57] McCarthy G. Puce A. Gore J. C. Allison T. (1997). Face-specific processing in the human fusiform gyrus. *J. Cogn. Neurosci.* 9 605–610. 10.1162/jocn.1997.9.5.605 23965119

[B58] McIntosh A. R. (1998). Understanding neural interactions in learning and memory using functional neuroimaging. *Ann. N. Y. Acad. Sci.* 855 556–571. 10.1111/j.1749-6632.1998.tb10625.x 9929651

[B59] Mennes M. Kelly C. Zuo X. N. Di Martino A. Biswal B. B. Castellanos F. X. (2010). Inter-individual differences in resting-state functional connectivity predict task-induced BOLD activity. *Neuroimage* 50 1690–1701. 10.1016/j.neuroimage.2010.01.002 20079856 PMC2839004

[B60] Mobbs D. Weiskopf N. Lau H. C. Featherstone E. Dolan R. J. Frith C. D. (2006). The Kuleshov Effect: the influence of contextual framing on emotional attributions. *Soc. Cogn. Affect. Neurosci.* 1 95–106. 10.1093/scan/nsl014 17339967 PMC1810228

[B61] Molenberghs P. Cunnington R. Mattingley J. B. (2012). Brain regions with mirror properties: a meta-analysis of 125 human fMRI studies. *Neurosci. Biobehav. Rev.* 36 341–349. 10.1016/j.neubiorev.2011.07.004 21782846

[B62] Morrison I. Tipper S. P. Fenton-Adams W. L. Bach P. (2013). “Feeling” others’ painful actions: the sensorimotor integration of pain and action information. *Hum. Brain Mapp.* 34 1982–1998. 10.1002/hbm.22040 22451259 PMC3807605

[B63] Muller V. I. Habel U. Derntl B. Schneider F. Zilles K. Turetsky B. I. (2011). Incongruence effects in crossmodal emotional integration. *Neuroimage* 54 2257–2266. 10.1016/j.neuroimage.2010.10.047 20974266 PMC8007888

[B64] Nichols T. Brett M. Andersson J. Wager T. Poline J. B. (2005). Valid conjunction inference with the minimum statistic. *Neuroimage* 25 653–660. 10.1016/j.neuroimage.2004.12.005 15808966

[B65] Northoff G. (2013). What the brain’s intrinsic activity can tell us about consciousness? A tri-dimensional view. *Neurosci. Biobehav. Rev.* 37 726–738. 10.1016/j.neubiorev.2012.12.004 23253946

[B66] Northoff G. (2014). *Unlocking the Brain.* New York: Oxford University Press.

[B67] Northoff G. Bermpohl F. (2004). Cortical midline structures and the self. *Trends Cogn. Sci.* 8 102–107. 10.1016/j.tics.2004.01.004 15301749

[B68] Northoff G. Schneider F. Rotte M. Matthiae C. Tempelmann C. Wiebking C. (2009). Differential parametric modulation of self-relatedness and emotions in different brain regions. *Hum. Brain Mapp.* 30 369–382. 10.1002/hbm.20510 18064583 PMC6870760

[B69] Okon-Singer H. Lichtenstein-Vidne L. Cohen N. (2013). Dynamic modulation of emotional processing. *Biol. Psychol.* 92 480–491. 10.1016/j.biopsycho.2012.05.010 22676964

[B70] Penny W. D. Stephan K. E. Mechelli A. Friston K. J. (2004). Modelling functional integration: a comparison of structural equation and dynamic causal models. *Neuroimage* 23 S264–S274. 10.1016/j.neuroimage.2004.07.041 15501096

[B71] Pessoa L. Adolphs R. (2010). Emotion processing and the amygdala: from a ‘low road’ to ‘many roads’ of evaluating biological significance. *Nat. Rev. Neurosci.* 11 773–783. 10.1038/nrn2920 20959860 PMC3025529

[B72] Pitcher D. Garrido L. Walsh V. Duchaine B. C. (2008). Transcranial magnetic stimulation disrupts the perception and embodiment of facial expressions. *J. Neurosci.* 28 8929–8933. 10.1523/JNEUROSCI.1450-08.2008 18768686 PMC6670866

[B73] Pourtois G. Spinelli L. Seeck M. Vuilleumier P. (2010). Temporal precedence of emotion over attention modulations in the lateral amygdala: Intracranial ERP evidence from a patient with temporal lobe epilepsy. *Cogn. Affect. Behav. Neurosci.* 10 83–93. 10.3758/CABN.10.1.83 20233957

[B74] Power J. D. Mitra A. Laumann T. O. Snyder A. Z. Schlaggar B. L. Petersen S. E. (2014). Methods to detect, characterize, and remove motion artifact in resting state fMRI. *Neuroimage* 84 320–341. 10.1016/j.neuroimage.2013.08.048 23994314 PMC3849338

[B75] Protzner A. B. McIntosh A. R. (2006). Testing effective connectivity changes with structural equation modeling: what does a bad model tell us? *Hum. Brain Mapp.* 27 935–947. 10.1002/hbm.20233 16929548 PMC6871338

[B76] Roy A. K. Shehzad Z. Margulies D. S. Kelly A. M. Uddin L. Q. Gotimer K. (2009). Functional connectivity of the human amygdala using resting state fMRI. *Neuroimage* 45 614–626. 10.1016/j.neuroimage.2008.11.030 19110061 PMC2735022

[B77] Rozzi S. Ferrari P. F. Bonini L. Rizzolatti G. Fogassi L. (2008). Functional organization of inferior parietal lobule convexity in the macaque monkey: electrophysiological characterization of motor, sensory and mirror responses and their correlation with cytoarchitectonic areas. *Europ. J. Neurosci.* 28 1569–1588. 10.1111/j.1460-9568.2008.06395.x 18691325

[B78] Saygin Z. M. Osher D. E. Augustinack J. Fischl B. Gabrieli J. D. (2011). Connectivity-based segmentation of human amygdala nuclei using probabilistic tractography. *Neuroimage* 56 1353–1361. 10.1016/j.neuroimage.2011.03.006 21396459 PMC3102511

[B79] Saygin Z. M. Osher D. E. Koldewyn K. Reynolds G. Gabrieli J. D. Saxe R. R. (2012). Anatomical connectivity patterns predict face selectivity in the fusiform gyrus. *Nat. Neurosci.* 15 321–327. 10.1038/nn.3001 22197830 PMC3267901

[B80] Schilbach L. Eickhoff S. B. Rotarska-Jagiela A. Fink G. R. Vogeley K. (2008). Minds at rest? Social cognition as the default mode of cognizing and its putative relationship to the “default system” of the brain. *Consciousn. Cogn.* 17 457–467. 10.1016/j.concog.2008.03.013 18434197

[B81] Schneider F. Bermpohl F. Heinzel A. Rotte M. Walter M. Tempelmann C. (2008). The resting brain and our self: self-relatedness modulates resting state neural activity in cortical midline structures. *Neurosci.* 157 120–131. 10.1016/j.neuroscience.2008.08.014 18793699

[B82] Schurz M. Radua J. Aichhorn M. Richlan F. Perner J. (2014). Fractionating theory of mind: a meta-analysis of functional brain imaging studies. *Neurosci. Biobehav. Rev.* 42 9–34. 10.1016/j.neubiorev.2014.01.009 24486722

[B83] Spreng R. N. Grady C. L. (2010). Patterns of brain activity supporting autobiographical memory, prospection, and theory of mind, and their relationship to the default mode network. *J. Cogn. Neurosci.* 22 1112–1123. 10.1162/jocn.2009.21282 19580387

[B84] Svoboda E. McKinnon M. C. Levine B. (2006). The functional neuroanatomy of autobiographical memory: a meta-analysis. *Neuropsychologia* 44 2189–2208. 10.1016/j.neuropsychologia.2006.05.023 16806314 PMC1995661

[B85] Talairach J. Tournoux P. (1988). *Co-planar Stereotaxic Atlas of the Human Brain. 3-Dimensional Proportional System: An Approach to Cerebral Imaging.* 1st Edn, Stuttgart: Thieme.

[B86] Touroutoglou A. Bickart K. C. Barrett L. F. Dickerson B. C. (2014). Amygdala task-evoked activity and task-free connectivity independently contribute to feelings of arousal. *Hum. Brain Mapp.* 35 5316–5327. 10.1002/hbm.22552 24862171 PMC4335688

[B87] van der Gaag C. Minderaa R. B. Keysers C. (2007). The BOLD signal in the amygdala does not differentiate between dynamic facial expressions. *Soc. Cogn. Affect. Neurosci.* 2 93–103. 10.1093/scan/nsm002 18985128 PMC2555450

[B88] van der Meer L. Costafreda S. Aleman A. David A. S. (2010). Self-reflection and the brain: a theoretical review and meta-analysis of neuroimaging studies with implications for schizophrenia. *Neurosci. Biobehav. Rev.* 34 935–946. 10.1016/j.neubiorev.2009.12.004 20015455

[B89] Van Dijk K. R. Hedden T. Venkataraman A. Evans K. C. Lazar S. W. Buckner R. L. (2010). Intrinsic functional connectivity as a tool for human connectomics: theory, properties, and optimization. *J. Neurophysiol.* 103 297–321. 10.1152/jn.00783.2009 19889849 PMC2807224

[B90] Van Overwalle F. Baetens K. (2009). Understanding others’ actions and goals by mirror and mentalizing systems: a meta-analysis. *Neuroimage* 48 564–584. 10.1016/j.neuroimage.2009.06.009 19524046

[B91] Vogt B. A. Vogt L. Laureys S. (2006). Cytology and functionally correlated circuits of human posterior cingulate areas. *Neuroimage* 29 452–466. 10.1016/j.neuroimage.2005.07.048 16140550 PMC2649771

[B92] Vuilleumier P. Pourtois G. (2007). Distributed and interactive brain mechanisms during emotion face perception: evidence from functional neuroimaging. *Neuropsychologia* 45 174–194. 10.1016/j.neuropsychologia.2006.06.003 16854439

[B93] Vuilleumier P. Richardson M. P. Armony J. L. Driver J. Dolan R. J. (2004). Distant influences of amygdala lesion on visual cortical activation during emotional face processing. *Nat. Neurosci.* 7 1271–1278. 10.1038/nn1341 15494727

[B94] Wang S. Tudusciuc O. Mamelak A. N. Ross I. B. Adolphs R. Rutishauser U. (2014). Neurons in the human amygdala selective for perceived emotion. *Proc. Nat. Acad. Sci. U.S.A.* 111 E3110–E3119. 10.1073/pnas.1323342111 PMC4121793 24982200

